# Women Hospitalized for Acute on Chronic Decompensated Systolic Heart Failure Receive Less Furosemide Compared to Men

**DOI:** 10.1155/2019/1505142

**Published:** 2019-09-12

**Authors:** Tyler P. Rasmussen, Noah N. Williford, Christopher DeZorzi, Aziz Hammoud, Brenden J. Boyle, Yunshu Zhou, Patrick Ten Eyck, Milena A. Gebska

**Affiliations:** ^1^Department of Internal Medicine, Carver College of Medicine, University of Iowa, Iowa City, IA 52242, USA; ^2^Division of Cardiovascular Medicine, Carver College of Medicine, University of Iowa, Iowa City, IA 52242, USA; ^3^Institute for Clinical and Translational Science, University of Iowa, Iowa City, IA 52242, USA

## Abstract

The cumulative incidence of systolic heart failure is similar in men and women. However, major prognostic differences exist between genders. We sought to measure gender differences in furosemide prescribing patterns for patients with preexisting heart failure with reduced ejection fraction (HFrEF) admitted with Stage C acute decompensation, regardless of the underlying cause. We conducted a single-center retrospective analysis of patients admitted between 2015 and 2018 for acute on chronic decompensated HFrEF. Primary outcomes were differences in initial furosemide dose, total dose over the first 24 hours of hospitalization, and total dose during the entire hospitalization between women and men. Secondary outcomes included acute kidney injury (AKI), intubation, noninvasive ventilation (NIV), and in-hospital 30-day and 1-year mortality. We studied 434 patients (31% female) with similar baseline characteristics. Females received significantly less furosemide compared to men for the initial dose, over the first 24 hours, and throughout their hospitalization. However, AKI was more prevalent in women versus men (*p*=0.008). Females admitted for acute on chronic decompensated HFrEF receive significantly less furosemide when compared to men, but developed more AKI prior to discharge.

## 1. Introduction

Cardiovascular disease remains the leading cause of death for both men and women in western societies and is a major healthcare burden with 4 million hospitalizations attributed to heart failure annually in the United States [1]. Heart failure affects approximately 1-2% of the population in developed countries [2] and prevalence exceeds 10% in septuagenarians [3].

Women with heart failure as compared to men with heart failure are less likely to be discharged on guideline-directed medical therapy [1], receive implantable cardioverter-defibrillators [4], undergo cardiac resynchronization therapy [5], and experience longer admission times [1] when identified as having decompensated heart failure. Gender differences related to presenting symptoms of acute coronary syndromes and coronary artery disease [6–8], arrhythmias [9], and heart failure [10–12] lead to a delay in recognition and optimal treatment in women compared to men. Symptomatic congestion from heart failure should be treated with diuretics irrespective of additional therapies or gender in patients admitted with Stage C acute decompensated heart failure [13]. Previous studies that included subjects with both preserved and reduced ejection fraction heart failure indicated that women receive similar regimens of diuretics compared to men [14]. Additionally, women as compared to men showed similar responses to intensive medical therapy when presenting with advanced decompensated HF (ejection fraction (EF) <20%; cardiac index <2.4 L/min/m^2^) [15]. However, specific differences in loop diuretic administration were not tested. Gender-specific differences in torsemide pharmacokinetics have been described in the literature [16], and animal studies have suggested a similar phenomenon with furosemide [17]. The clinical significance of this potential pharmacokinetic gender difference remains unclear.

The question remains whether initial furosemide prescribing patterns to overcome diuretic resistance in patients with acute on chronic decompensated heart failure with reduced ejection fraction (HFrEF) differ between sexes when patients are admitted to the hospital. And furthermore, to what extent this potential difference affects length of stay, renal injury, respiratory decompensation, and mortality remains untested.

We sought to determine whether gender differences in furosemide prescribing patterns exist in patients admitted to the hospital with acute decompensated HFrEF, irrespective of the underlying cause. Establishing whether such difference exists could prompt further investigation into both the cause of differential treatment and methods to mitigate it. Another important aspect of our study was to determine whether the potential difference in initial furosemide prescription strength led to acute kidney injury (AKI), respiratory failure, and 30 day mortality. Identifying a difference in any of these clinical outcomes would suggest that standardizing diuretic dosing to men and women admitted with acute decompensated HFrEF could abate disparate outcomes caused by gender.

## 2. Methods

### 2.1. Data Source

All data were collected from the University of Iowa electronic medical record.

### 2.2. Study Population

This is a single-center retrospective analysis of all patients with heart failure with reduced ejection fraction, defined as LVEF ≤40%, irrespective of the underlying cause, admitted to our institution for acute on chronic decompensation between July 1, 2015, and June 30, 2018. Inclusion criteria were Stage C acutely decompensated heart failure, transthoracic echocardiogram- (TTE-) documented LVEF ≤40% prior to or during admission, age greater than 18, and administration of furosemide (cumulative intravenous and *per os* doses). Patients were excluded if they did not have a TTE available within a year prior to admission, if TTE demonstrated LVEF >40%, if bumetanide or torsemide were administered during the hospitalization, if they had end-stage renal disease, or if they were diagnosed with cardiogenic shock during the hospitalization. In addition, we did not analyze cause of cardiomyopathy, presence of atrial fibrillation, and cardiovascular risk factors as they do not impact clinical decision making about dosing diuretics.

### 2.3. Outcome Measures and Definitions

The primary outcome was the difference in furosemide dosing in women, as compared to men initially, over the first 24 hours of hospitalization, or over the entire length of stay (LOS). Secondary outcomes were rates of AKI, endotracheal intubation, noninvasive ventilation (NIV), and in-hospital 30-day and 1-year mortality. We relied on ICD coding to identify patients who developed AKI during the hospitalization.

### 2.4. Statistics

We employed the Wilcoxon rank sum test to compare initial, 24 hour, and total furosemide doses between men and women. Comparisons were made on the raw doses as well as normalized values using glomerular filtration rate (GFR), N-terminal-prohormone brain natriuretic peptide (NT-proBNP), BMI, and ejection fraction (EF). Normalized values are the raw values divided by the normalizing variable. Our type I error rate for this analysis is *α* = 0.05, but because we are making 12 comparisons, we adjusted our cutoff for claiming significance. Using the Bonferroni correction, comparisons with *p* values ≤0.05/12 = 0.0042 were deemed significant. We used logistic regression to fit multivariate models relating sex, dose, and their interaction to our secondary outcomes while controlling for age, EF, GFR, supplemental oxygen, systolic blood pressure (SBP), diastolic blood pressure (DBP), and NT-proBNP. Estimates for predictor odds ratios and 95% confidence intervals were calculated along with the *p* values. Statistical analyses were performed using SAS 9.4 (SAS Institute, Cary, NC, USA).

## 3. Results

We identified 1,437 subjects who were admitted with a diagnosis of acute on chronic decompensated HFrEF. Of these, 708 subjects were excluded because of torsemide or bumetanide administration, end-stage renal disease, or diagnosis of cardiogenic shock. An additional 139 subjects had no documented left ventricular systolic function prior to admission, and 156 subjects had documented EF >40%, all of which were also excluded. A total of 434 subjects remained eligible for study. Compared with men, women (31%) had similar baseline characteristics except for slightly lower GFR in women (Table 1).

**Figure 1:**
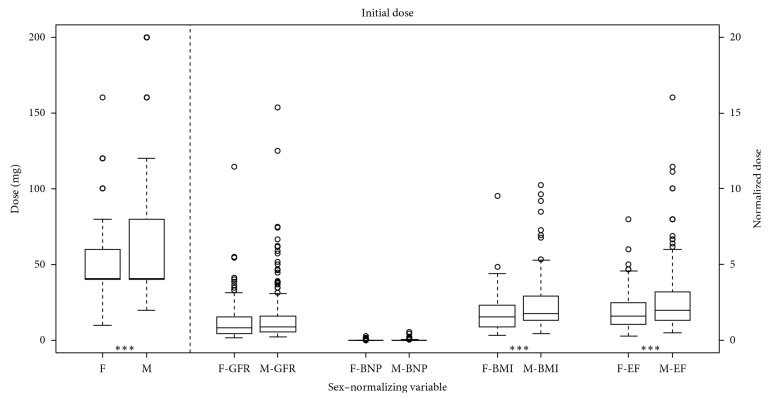
Median initial furosemide dose in milligrams for females (F) and males (M) with boxplots representing the interquartile range. Values left of the dashed line are absolute. Values right of the dashed line are normalized to GFR, NT-proBNP, BMI, and EF, respectively. ^*∗∗∗*^*p* < 0.001.

**Figure 2:**
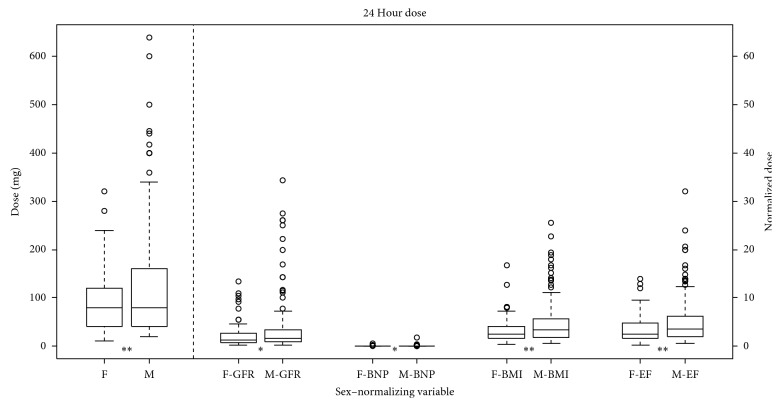
Median furosemide dose for first 24 hours of hospitalization in milligrams for females (F) and males (M) with boxplots representing the interquartile range. Values left of the dashed line are absolute. Values right of the dashed line are normalized to GFR, BNP, BMI, and EF, respectively. ^*∗*^*p* < 0.05; ^*∗∗*^*p* < 0.01.

**Figure 3:**
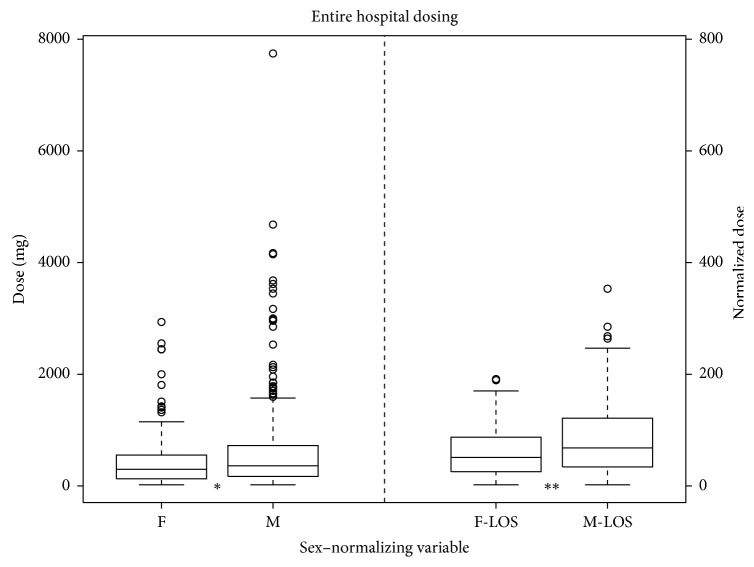
Median furosemide dose for entire hospitalization in milligrams for females (F) and males (M) with boxplots representing the interquartile range. Values left of the dashed line are absolute. Values right of the dashed line are normalized to LOS. ^*∗*^*p* < 0.05; ^*∗∗*^*p* < 0.01.

We found that women received significantly less furosemide than men initially (*p*=0.0007) and when normalized to BMI (*p*=0.0005) or EF (*p*=0.0002). There was no significant difference detected when normalizing to GFR (*p*=0.19) or NT-proBNP (*p*=0.09) (Figure 1). Furthermore, women received less furosemide during the first 24 hours of hospitalization (*p*=0.0025), and this significance remained when normalized to GFR (*p* < 0.05), NT-proBNP (*p* < 0.05), BMI (*p*=0.001), and EF (*p*=0.002) (Figure 2). The total dose of furosemide when normalized to the LOS was less in women as compared to men (*p*=0.0015) (Figure 3).

We performed a subgroup analysis of patients with EF less than 30% and found similar differences in furosemide dosing when compared to our entire cohort. However, in this subgroup with severely impaired left ventricular systolic function, the amount of furosemide prescribed to women when compared to men over an entire hospitalization was not statistically different when normalized to LOS.

We compared rates of AKI, in-hospital death, 30 day mortality, 1 year mortality, intubation, and NIV. When initial furosemide dose was normalized to admission NT-proBNP, women were more likely than men to develop an AKI (*p*=0.0081). In-hospital death (*p*=0.2221), 30 day mortality (*p*=0.4106), 1 year mortality (*p*=0.2463), intubation (*p*=0.2421) and NIV (*p*=0.0994) were not statistically different between sexes, suggesting that furosemide dosing used to treat diuretic resistance plays a limited role in clinical outcomes, at least in the population we studied.

After adjusting for age, EF, GFR, oxygen, SBP, DBP, and NT-proBNP, women developed significantly more AKI compared to men (*p*=0.0081). The remainder of the outcome variables were similar between genders.

## 4. Discussion

Prior to this study, it was unknown whether women when compared to men with preexisting HFrEF admitted to the hospital with acutely decompensated heart failure received similar or different doses of furosemide to treat symptoms and overcome diuretic resistance. Recent studies have shown gender bias with regard to treatment strategies that lead to delayed interventions in women with chronic heart failure compared to men [10–12]. Our study showed that women were prescribed less furosemide upon admission, over the first 24 hours of stay, and over the entire hospitalization period. However, the relative lower doses did not lead to meaningful clinical disparities between genders, other than an association with higher AKI rates in women.

Differences in furosemide pharmacodynamics between genders may contribute to a higher rate of worsening renal function in women. Possible explanations for a gender bias are different phenotypes of hypervolemia in men and women and differences in physical examination practices. Our data beg for more prospective research to improve standardization of heart failure treatment between both genders. We acknowledge that retrospective study design, data collection using ICD coding, and small sample size from a single center limit our study interpretation.

We were surprised that women who received less furosemide compared to matched men were more likely to develop an AKI during hospitalization (*p*=0.008). We initially hypothesized that men would be more prone to AKI due to higher furosemide administration. This finding further highlights physiological and compensatory gender differences that should be considered in clinical practice. Perhaps, women received inadequate furosemide doses to relieve congestion in the renal efferent arterioles, thereby preventing recovery of renal function. An alternate explanation could be differences in furosemide pharmacokinetics in women versus men that portend them toward renal injury when high doses of loop diuretics are prescribed. Finally, women may require different strategies to overcome diuretic resistance compared with men. We recognize that using ICD coding to identify patients with AKI is a limitation of our study. Ultimately, a prospective multicenter study designed to analyze prespecified changes in renal function from baseline values would be necessary to determine whether women are more likely than men to develop an AKI when admitted for acutely decompensated systolic heart failure and could elucidate the mechanisms underlying our findings.

## 5. Conclusions

In our single-center retrospective review, we found that women with preexisting HFrEF were underrepresented at the time of admission for acute decompensation, yet they received significantly lower doses of furosemide as compared to men at the time of symptom recognition and during entire hospitalization. Interestingly, these differences in diuretic dosing were associated with higher rates for AKI in women but did not prolong LOS or cause increased mortality. Ultimately, a prospective randomized study is desired to provide greater understanding of an optimal diuretic management in women with acute on chronic decompensated systolic heart failure to overcome diuretic resistance [12].

## Figures and Tables

**Table 1:** Baseline characteristics between female and male subjects.

Variable	Female (*N* = 135)			Male (*N* = 298)			*p* value
	Q1	Median	Q3	Q1	Median	Q3	
Age	57	68	79	58	65	77	0.3629
BMI (kg/m^2^)	23.6	26.8	32.9	23.6	26.8	32	0.7566
Systolic BP (mmHg)	106	116	130	102	113	127	0.3132
Diastolic BP (mmHg)	56	65	75	59	67	78.5	0.0562
Troponin T (ng/mL)	0	0	0.1	0	0	0.1	0.2584
NT-proBNP (pg/mL)	3341	7293	18608	3357	7318	14334	0.6080
EF (%)	20	25	32.5	20	25	33	0.5270
GFR (ml/min)	35	52	70	43	56	75	0.0389
Oxygen requirement		13.3%			17.1%		0.3952

Q1 and Q3 represent the cutoffs for 1^st^ and 3^rd^ quartiles of the interquartile range.

## Data Availability

The data used to support the findings of this study are available from the corresponding author upon request.
